# *N*-Acetonylsaccharin

**DOI:** 10.1107/S1600536809030773

**Published:** 2009-08-19

**Authors:** Matloob Ahmad, Hamid Latif Siddiqui, Muhammad Azam, Waseeq Ahmad Siddiqui, Masood Parvez

**Affiliations:** a Institute of Chemistry, University of the Punjab, Lahore 54590, Pakistan; b Institute of Biochemistry, University of Balochistan, Quetta, Pakistan; c Department of Chemistry, University of Sargodha, Sargodha, Pakistan; d Department of Chemistry, The University of Calgary, 2500 University Drive NW, Calgary, Alberta, T2N 1N4, Canada

## Abstract

In the title compound [systematic name: 2-(2-oxoprop­yl)-1,2-benzothia­zol-3(2*H*)-one 1,1-dioxide], C_10_H_9_NO_4_S, the benzo­thia­zole unit is essentially planar [maximum deviation = 0.0490 (9) Å for the S atom] and the oxopropyl group is inclined at an angle 75.61 (8)° with respect to its mean plane. In the crystal, mol­ecules are held together by weak inter­molecular C—H⋯O non-classical hydrogen bonds, resulting in centrosymmetric dimeric units, forming 14-membered ring systems which may be described as *R*_2_^2^(14) ring motifs. Moreover, mol­ecules lying about inversion centers show π–π inter­actions, with centroid–centroid separations between the benzene rings of 3.676 (2) Å.

## Related literature

For the crystal structure of a benzothia­zine, see: Ahmad *et al.* (2008 ▶). For the biological activity of sacharine derivatives, see: Kapui *et al.* (2003 ▶); Singh *et al.* (2007 ▶); Vaccarino *et al.* (2007 ▶). For graph-set notation of ring motifs, see: Bernstein *et al.* (1994 ▶).
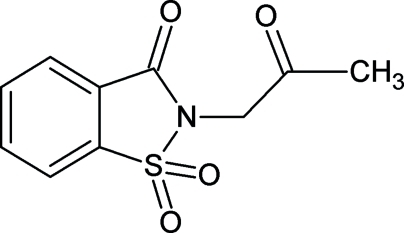


## Experimental

### 

#### Crystal data

C_10_H_9_NO_4_S
*M* *_r_* = 239.24
Monoclinic, 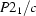

*a* = 7.475 (3) Å
*b* = 8.975 (4) Å
*c* = 15.923 (7) Å
β = 101.028 (18)°
*V* = 1048.5 (8) Å^3^
*Z* = 4
Mo *K*α radiation
μ = 0.31 mm^−1^
*T* = 200 K
0.12 × 0.12 × 0.06 mm

#### Data collection

Nonius KappaCCD diffractometer
Absorption correction: multi-scan (*SORTAV*; Blessing, 1997 ▶) *T*_min_ = 0.964, *T*_max_ = 0.982
3984 measured reflections
2382 independent reflections
2106 reflections with *I* > 2σ(*I*)
*R*_int_ = 0.023

#### Refinement

*R*[*F* ^2^ > 2σ(*F* ^2^)] = 0.041
*wR*(*F* ^2^) = 0.110
*S* = 1.04
2382 reflections
146 parameters
H-atom parameters constrained
Δρ_max_ = 0.30 e Å^−3^
Δρ_min_ = −0.41 e Å^−3^

### 

Data collection: *COLLECT* (Hooft, 1998 ▶); cell refinement: *DENZO* (Otwinowski & Minor, 1997 ▶); data reduction: *SCALEPACK* (Otwinowski & Minor, 1997 ▶); program(s) used to solve structure: *SHELXS97* (Sheldrick, 2008 ▶); program(s) used to refine structure: *SHELXL97* (Sheldrick, 2008 ▶); molecular graphics: *ORTEP-3 for Windows* (Farrugia, 1997 ▶); software used to prepare material for publication: *SHELXL97*.

## Supplementary Material

Crystal structure: contains datablocks global, I. DOI: 10.1107/S1600536809030773/si2191sup1.cif

Structure factors: contains datablocks I. DOI: 10.1107/S1600536809030773/si2191Isup2.hkl

Additional supplementary materials:  crystallographic information; 3D view; checkCIF report

## Figures and Tables

**Table 1:** Hydrogen-bond geometry (Å, °)

*D*—H⋯*A*	*D*—H	H⋯*A*	*D*⋯*A*	*D*—H⋯*A*
C10—H10*B*⋯O2^i^	0.98	2.57	3.324 (3)	134

Symmetry code: (i) 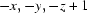.

**Fig. 1.**
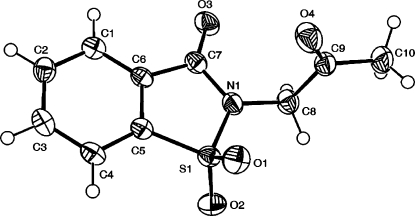
ORTEP-3 (Farrugia, 1997) drawing of (I) with displacement ellipsoids plotted at 50% probability level.

**Fig. 2.**
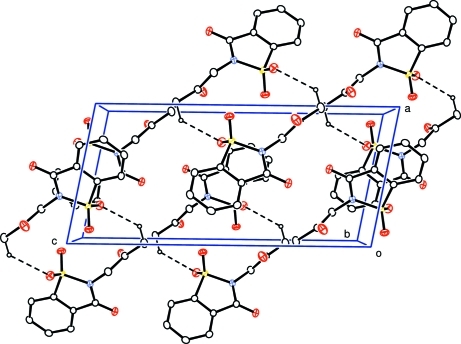
Unit cell of (I) showing dimers of molecules formed by C—H···O interactions; H-atoms not involved in H-bonding interactions have been excluded.

## supplementary crystallographic information

### Comment

Saccharine derivatives are extensively reported in the literature for their diverse range of biological activities like cyclooxygenase-2 (COX-2) inhibitors (Singh *et al.* 2007), analgesic (Vaccarino *et al.* 2007), human leucocyte elastase (HLE) inhibitors (Kapui *et al.* 2003). In continuation to our project to explore potentially biologically active derivatives of benzothiazines (Ahmad *et al.* 2008), we herein report the crystal structure of the title compound, (I), in this paper.

The structure of the title compound is depicted in Figure 1. The benzothiazol moiety (C1—C7/N1/S1) is essentailly planar with maximum deviation observed for S1 (0.0490 (9) Å) and the oxopropyl group (C8/C9/C10/O4) forms an angle 75.61 (8)° with the mean-plane of the former. The molecular dimensions in (I) agree with the corresponding molecular dimensions reported for a closely related compound (Ahmad *et al.* 2008). In the crystal structure, the molecules of (I) are held together by rather weak intermolecular C—H···O type non-classical hydrogen bonds resulting in dimeric units about inversion centers, forming fourteen membered ring systems which may be described in terms of graph set notation (Bernstein *et al.* 1994) as R_2_^2^(14) ring motif; details are given in Table 1 and Figure 2. The molecules lying about inversion centers show π-π interactions with the separation between the centroids of the benzene rings (C1–C6) which are related by the symmetry operation: 1-x, 1-y, 1-z, is 3.676 (2) Å (Spek, 2009); with perpendicular distance being 3.354 Å and the slippage of 1.504 Å.

### Experimental

Sodium saccharin (73.2 mmoles, 15.0 g) and chloroacetone (87.8 mmole, 7.0 ml) were added in a round bottom flask containing 30 ml of anhydrous DMF. The mixture was stirred under inert atmosphere for one hour at 393 K. The contents of the flask were poured in ice cold water. Brownish ppts. formed were filtered and washed with excess of water. Crystals suitable for XRD were grown in chloroform. Yield: 15.2 g, 87%; m.p. 386–387 K.

### Refinement

Though all the H atoms could be distinguished in the difference Fourier map the H-atoms were included at geometrically idealized positions and refined in riding-model approximation with the following constraints: C—H distances were set to 0.95–0.99 Å and *U*_iso_(H) = 1.2*U*_eq_(C). The final difference map was free of any chemically significant features.

### Crystal data

**Table:**

C_10_H_9_NO_4_S	*F*(000) = 496
*M**_r_* = 239.24	*D*_x_ = 1.516 Mg m^−^^3^
Monoclinic, *P*2_1_/*c*	Mo *K*α radiation, λ = 0.71073 Å
Hall symbol: -P 2ybc	Cell parameters from 3984 reflections
*a* = 7.475 (3) Å	θ = 3.4–27.5°
*b* = 8.975 (4) Å	µ = 0.31 mm^−^^1^
*c* = 15.923 (7) Å	*T* = 200 K
β = 101.028 (18)°	Block, colorless
*V* = 1048.5 (8) Å^3^	0.12 × 0.12 × 0.06 mm
*Z* = 4	

### Data collection

**Table:**

Nonius KappaCCD diffractometer	2382 independent reflections
Radiation source: fine-focus sealed tube	2106 reflections with *I* > 2σ(*I*)
graphite	*R*_int_ = 0.023
ω and φ scans	θ_max_ = 27.5°, θ_min_ = 3.4°
Absorption correction: multi-scan (*SORTAV*; Blessing, 1997)	*h* = −9→9
*T*_min_ = 0.964, *T*_max_ = 0.982	*k* = −8→11
3984 measured reflections	*l* = −20→20

### Refinement

**Table:**

Refinement on *F*^2^	Primary atom site location: structure-invariant direct methods
Least-squares matrix: full	Secondary atom site location: difference Fourier map
*R*[*F*^2^ > 2σ(*F*^2^)] = 0.041	Hydrogen site location: inferred from neighbouring sites
*wR*(*F*^2^) = 0.110	H-atom parameters constrained
*S* = 1.04	*w* = 1/[σ^2^(*F*_o_^2^) + (0.0444*P*)^2^ + 0.8524*P*] where *P* = (*F*_o_^2^ + 2*F*_c_^2^)/3
2382 reflections	(Δ/σ)_max_ = 0.005
146 parameters	Δρ_max_ = 0.30 e Å^−^^3^
0 restraints	Δρ_min_ = −0.41 e Å^−^^3^

### Special details

**Table:**

Geometry. All e.s.d.'s (except the e.s.d. in the dihedral angle between two l.s. planes) are estimated using the full covariance matrix. The cell e.s.d.'s are taken into account individually in the estimation of e.s.d.'s in distances, angles and torsion angles; correlations between e.s.d.'s in cell parameters are only used when they are defined by crystal symmetry. An approximate (isotropic) treatment of cell e.s.d.'s is used for estimating e.s.d.'s involving l.s. planes.
Refinement. Refinement of *F*^2^ against ALL reflections. The weighted *R*-factor *wR* and goodness of fit *S* are based on *F*^2^, conventional *R*-factors *R* are based on *F*, with *F* set to zero for negative *F*^2^. The threshold expression of *F*^2^ > σ(*F*^2^) is used only for calculating *R*-factors(gt) *etc*. and is not relevant to the choice of reflections for refinement. *R*-factors based on *F*^2^ are statistically about twice as large as those based on *F*, and *R*- factors based on ALL data will be even larger.

### Fractional atomic coordinates and isotropic or equivalent isotropic displacement parameters (Å^2^)

**Table:**

	*x*	*y*	*z*	*U*_iso_*/*U*_eq_	
S1	0.26215 (6)	0.14487 (5)	0.47133 (3)	0.02833 (15)	
O1	0.09274 (19)	0.22262 (17)	0.44859 (10)	0.0401 (4)	
O2	0.2659 (2)	−0.00422 (16)	0.44012 (10)	0.0406 (4)	
O3	0.5536 (2)	0.25086 (18)	0.68078 (9)	0.0397 (4)	
O4	0.1159 (2)	0.30470 (18)	0.66321 (11)	0.0504 (4)	
N1	0.3345 (2)	0.14693 (19)	0.57704 (10)	0.0319 (4)	
C1	0.7056 (3)	0.3839 (2)	0.53131 (12)	0.0306 (4)	
H1	0.7806	0.4139	0.5837	0.037*	
C2	0.7458 (3)	0.4271 (2)	0.45301 (13)	0.0335 (4)	
H2	0.8482	0.4894	0.4520	0.040*	
C3	0.6389 (3)	0.3809 (2)	0.37630 (13)	0.0355 (4)	
H3	0.6702	0.4113	0.3238	0.043*	
C4	0.4873 (3)	0.2911 (2)	0.37500 (12)	0.0317 (4)	
H4	0.4148	0.2579	0.3227	0.038*	
C5	0.4466 (2)	0.2519 (2)	0.45337 (12)	0.0267 (4)	
C6	0.5534 (2)	0.29595 (19)	0.53062 (11)	0.0263 (4)	
C7	0.4875 (3)	0.2339 (2)	0.60579 (12)	0.0295 (4)	
C8	0.2358 (3)	0.0693 (2)	0.63437 (13)	0.0350 (4)	
H8A	0.3243	0.0170	0.6788	0.042*	
H8B	0.1557	−0.0068	0.6014	0.042*	
C9	0.1209 (3)	0.1729 (2)	0.67757 (13)	0.0346 (4)	
C10	0.0182 (3)	0.1008 (3)	0.73859 (14)	0.0500 (6)	
H10A	0.0730	0.0039	0.7564	0.060*	
H10B	−0.1092	0.0867	0.7104	0.060*	
H10C	0.0235	0.1646	0.7890	0.060*	

### Atomic displacement parameters (Å^2^)

**Table:**

	*U*^11^	*U*^22^	*U*^33^	*U*^12^	*U*^13^	*U*^23^
S1	0.0268 (2)	0.0309 (2)	0.0263 (2)	0.00255 (17)	0.00270 (17)	−0.00209 (17)
O1	0.0271 (7)	0.0465 (8)	0.0446 (9)	0.0070 (6)	0.0015 (6)	0.0000 (7)
O2	0.0477 (9)	0.0311 (7)	0.0415 (8)	−0.0004 (6)	0.0049 (7)	−0.0067 (6)
O3	0.0419 (8)	0.0536 (9)	0.0221 (7)	−0.0003 (7)	0.0024 (6)	0.0009 (6)
O4	0.0612 (11)	0.0388 (8)	0.0582 (11)	0.0046 (8)	0.0288 (9)	0.0006 (7)
N1	0.0303 (8)	0.0411 (9)	0.0252 (8)	−0.0019 (7)	0.0077 (6)	−0.0004 (6)
C1	0.0278 (9)	0.0337 (9)	0.0289 (9)	0.0050 (7)	0.0021 (7)	−0.0009 (7)
C2	0.0286 (9)	0.0344 (10)	0.0393 (11)	0.0029 (8)	0.0105 (8)	0.0018 (8)
C3	0.0367 (10)	0.0430 (11)	0.0291 (10)	0.0065 (8)	0.0124 (8)	0.0051 (8)
C4	0.0334 (9)	0.0382 (10)	0.0230 (9)	0.0071 (8)	0.0043 (7)	−0.0016 (7)
C5	0.0251 (8)	0.0287 (8)	0.0259 (9)	0.0051 (7)	0.0040 (7)	−0.0006 (7)
C6	0.0278 (9)	0.0280 (8)	0.0229 (8)	0.0061 (7)	0.0043 (7)	−0.0008 (7)
C7	0.0290 (9)	0.0345 (9)	0.0249 (9)	0.0052 (7)	0.0045 (7)	0.0005 (7)
C8	0.0389 (10)	0.0345 (10)	0.0330 (10)	−0.0006 (8)	0.0103 (8)	0.0053 (8)
C9	0.0322 (10)	0.0435 (11)	0.0288 (10)	−0.0055 (9)	0.0075 (8)	0.0006 (8)
C10	0.0500 (13)	0.0703 (16)	0.0330 (12)	−0.0204 (12)	0.0159 (10)	−0.0010 (11)

### Geometric parameters (Å, °)

**Table:**

S1—O2	1.4295 (15)	C3—C4	1.388 (3)
S1—O1	1.4305 (15)	C3—H3	0.9500
S1—N1	1.6667 (18)	C4—C5	1.385 (3)
S1—C5	1.7485 (19)	C4—H4	0.9500
O3—C7	1.211 (2)	C5—C6	1.389 (3)
O4—C9	1.204 (3)	C6—C7	1.487 (3)
N1—C7	1.388 (3)	C8—C9	1.517 (3)
N1—C8	1.456 (2)	C8—H8A	0.9900
C1—C6	1.383 (3)	C8—H8B	0.9900
C1—C2	1.392 (3)	C9—C10	1.496 (3)
C1—H1	0.9500	C10—H10A	0.9800
C2—C3	1.389 (3)	C10—H10B	0.9800
C2—H2	0.9500	C10—H10C	0.9800
			
O2—S1—O1	116.35 (9)	C6—C5—S1	110.42 (14)
O2—S1—N1	109.71 (9)	C1—C6—C5	120.11 (17)
O1—S1—N1	110.53 (9)	C1—C6—C7	127.31 (17)
O2—S1—C5	112.90 (9)	C5—C6—C7	112.55 (16)
O1—S1—C5	112.22 (9)	O3—C7—N1	123.44 (18)
N1—S1—C5	92.60 (8)	O3—C7—C6	127.64 (18)
C7—N1—C8	123.13 (17)	N1—C7—C6	108.91 (16)
C7—N1—S1	115.29 (13)	N1—C8—C9	112.95 (16)
C8—N1—S1	121.49 (14)	N1—C8—H8A	109.0
C6—C1—C2	118.03 (18)	C9—C8—H8A	109.0
C6—C1—H1	121.0	N1—C8—H8B	109.0
C2—C1—H1	121.0	C9—C8—H8B	109.0
C3—C2—C1	121.19 (19)	H8A—C8—H8B	107.8
C3—C2—H2	119.4	O4—C9—C10	123.2 (2)
C1—C2—H2	119.4	O4—C9—C8	121.05 (18)
C4—C3—C2	121.16 (18)	C10—C9—C8	115.78 (19)
C4—C3—H3	119.4	C9—C10—H10A	109.5
C2—C3—H3	119.4	C9—C10—H10B	109.5
C5—C4—C3	116.97 (18)	H10A—C10—H10B	109.5
C5—C4—H4	121.5	C9—C10—H10C	109.5
C3—C4—H4	121.5	H10A—C10—H10C	109.5
C4—C5—C6	122.52 (18)	H10B—C10—H10C	109.5
C4—C5—S1	127.07 (15)		
			
O2—S1—N1—C7	120.12 (15)	C2—C1—C6—C7	178.29 (17)
O1—S1—N1—C7	−110.26 (15)	C4—C5—C6—C1	0.9 (3)
C5—S1—N1—C7	4.66 (15)	S1—C5—C6—C1	−179.02 (13)
O2—S1—N1—C8	−63.36 (17)	C4—C5—C6—C7	−177.06 (16)
O1—S1—N1—C8	66.26 (17)	S1—C5—C6—C7	3.04 (19)
C5—S1—N1—C8	−178.83 (15)	C8—N1—C7—O3	1.2 (3)
C6—C1—C2—C3	−1.4 (3)	S1—N1—C7—O3	177.61 (15)
C1—C2—C3—C4	0.6 (3)	C8—N1—C7—C6	179.96 (16)
C2—C3—C4—C5	0.9 (3)	S1—N1—C7—C6	−3.59 (19)
C3—C4—C5—C6	−1.7 (3)	C1—C6—C7—O3	1.1 (3)
C3—C4—C5—S1	178.21 (14)	C5—C6—C7—O3	178.91 (18)
O2—S1—C5—C4	63.10 (19)	C1—C6—C7—N1	−177.59 (17)
O1—S1—C5—C4	−70.77 (19)	C5—C6—C7—N1	0.2 (2)
N1—S1—C5—C4	175.78 (17)	C7—N1—C8—C9	74.6 (2)
O2—S1—C5—C6	−117.01 (13)	S1—N1—C8—C9	−101.65 (19)
O1—S1—C5—C6	109.12 (14)	N1—C8—C9—O4	0.4 (3)
N1—S1—C5—C6	−4.32 (14)	N1—C8—C9—C10	−178.98 (18)
C2—C1—C6—C5	0.7 (3)		

### Hydrogen-bond geometry (Å, °)

**Table:**

*D*—H···*A*	*D*—H	H···*A*	*D*···*A*	*D*—H···*A*
C10—H10B···O2^i^	0.98	2.57	3.324 (3)	134

Symmetry codes: (i) −*x*, −*y*, −*z*+1.
